# ^18^F-FDG uptake of visceral adipose tissue on preoperative PET/CT as a predictive marker for breast cancer recurrence

**DOI:** 10.1038/s41598-022-25540-4

**Published:** 2022-12-06

**Authors:** Hyun Jeong Kim, Dooreh Kim, Soong June Bae, Sung Gwe Ahn, Joon Jeong, Woo-Chan Park, Young Hoon Ryu, Tae Joo Jeon, Yangkyu Lee, Yoon Jin Cha, Chang Ik Yoon

**Affiliations:** 1 Department of Nuclear Medicine, Yongin Severance Hospital, Yonsei University College of Medicine, Yongin, Republic of Korea; 2 Division of Breast Surgery, Department of Surgery, College of Medicine, Seoul St Mary’s Hospital, The Catholic University of Korea, 222 Banpo-daero, Seocho-Gu, Seoul, 06591 Republic of Korea; 3 Department of Surgery, Gangnam Severance Hospital, Yonsei University College of Medicine, Seoul, Republic of Korea; 4 Institute for Breast Cancer Precision Medicine, Yonsei University College of Medicine, Seoul, Republic of Korea; 5 Department of Nuclear Medicine, Gangnam Severance Hospital, Yonsei University College of Medicine, Seoul, Republic of Korea; 6 Department of Pathology, Gangnam Severance Hospital, Yonsei University College of Medicine, Eonju-Ro 211, Gangnam-Gu, Seoul, 06273 Republic of Korea

**Keywords:** Breast cancer, Breast cancer

## Abstract

Glucose utilization by visceral adipose tissue (VAT) reflects inflammatory activity, which also promotes tumor growth and carcinogenesis. The effect of metabolically active VAT on survival outcomes in breast cancer is unknown. We investigated survival outcomes in patients with breast cancer based on the standardized uptake value (SUV) of VAT (SUVmean-VAT) using ^18^F-fluorodeoxyglucose positron emission tomography/computed tomography (^18^F-FDG PET/CT). A total of 148 patients with breast cancer were divided into high- and low groups according to their SUVmean-VAT and SUVmax-tumor. Clinical characteristics and survival outcomes were compared between the groups. High SUVmean-VAT was associated with poor recurrence-free survival (RFS; hazard ratio [HR], 2.754; 95% confidence interval [CI], 1.090–6.958, *p* = 0.032) and distant metastasis-free survival (DMFS; HR, 3.500; 95% CI, 1.224–10.01, *p* = 0.019). Multivariate analysis showed that high SUVmean-VAT was a significant factor for poor RFS and poor DMFS (*p* = 0.023 and 0.039, respectively). High SUVmax-tumor was significantly associated with short RFS (*p* = 0.0388). Tumors with a high SUV tended to have a short DMFS, although the difference was not significant (*p* = 0.0718). Our findings showed that upregulated glucose metabolism in the VAT measured using ^18^F-FDG PET/CT may be a prognostic biomarker for adverse outcomes in breast cancer.

## Introduction

Breast cancer is a leading cause of cancer-related death in women worldwide^1^. Despite advances in early cancer detection and the multimodal treatment approach, systemic relapse of breast cancer still occurs. Recent studies have reported the independent association between tumor relapse and elevation of serum inflammatory biomarkers, including C-reactive protein (CRP), interleukin-6, and serum amyloid A; this suggests that systemic inflammation affects breast cancer recurrence^2^. The inflammatory compartment of the tumor microenvironment is deeply involved in breast cancer progression and metastasis^3^, which includes tumor-associated macrophages, matrix metalloproteinase, sphingosine 1-phosphate (S1P), and CRP^4–6^. Particularly, S1P and CRP promote breast cancer metastasis by influencing tumor cell invasion^7^.

^18^Fluorodeoxyglucose (^18^F-FDG) uptake by positron emission tomography (PET)/computed tomography (CT) is higher in areas with high metabolic activities, such as inflammatory reaction sites and tumors. Pahk et al. showed that ^18^F-FDG uptake in visceral adipose tissue (VAT) was positively correlated with carotid artery inflammation and the severity of coronary artery disease in humans^8^. Furthermore, they reported a correlation between the relative metabolic activity of VAT, estimated as the maximum standardized uptake value (SUVmax) divided by the SUVmax of subcutaneous adipose tissue (SAT), and frequent lymph node metastasis in luminal breast cancer in postmenopausal patients^9^. Based on previous study results^8,9^, we speculated that an increased SUV of VAT (SUVmean-VAT) reflects the systemic inflammatory status, which can be related to the clinical outcome of breast cancer. In this study, we aimed to measure the SUVmean-VAT and maximum SUV of the tumors (SUVmax-tumor) in patients with breast cancer to analyze their clinical implications.

## Results

### Baseline characteristics based on the ^18^F-FDG uptake of VAT

**Figure 1:**
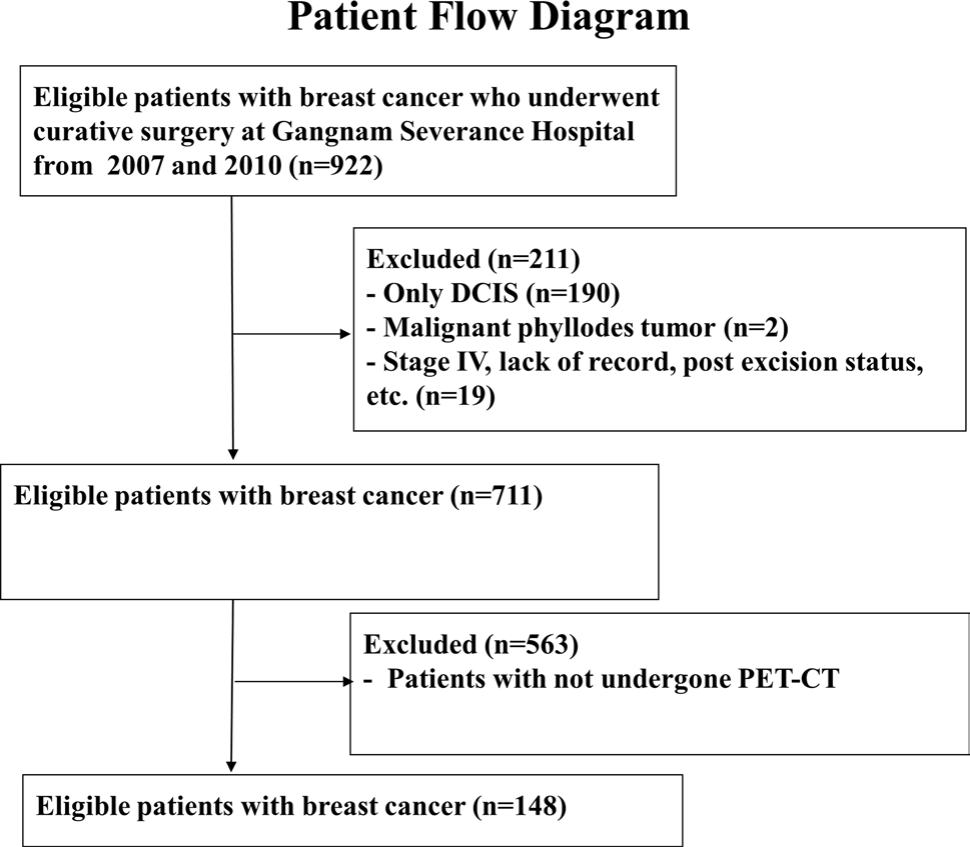
Patient flow diagram showing the study design. DCIS: ductal carcinoma in situ, PET/CT: positron emission tomography/computed tomography.

In total, 922 patients with breast cancer were screended for this study. We excluded 211 patients with de novo stage IV breast cancer, ductal carcinoma in situ alone, and etc (Figure 1). In addition, 563 patients had no PET-CT exam and thus were excluded. Data from 148 patients were analyzed in this study. Figure 1 shows the patient flow diagram. The median age of the study population was 49 years (range: 26–87 years). The average SUVmax-tumor for all the patients was 4.49 ± 3.67, and the median value was 3.30 (Supplementary Fig. 1a). The SUVmean-VAT was 0.42 ± 0.11, and the median value was 0.41 (Supplementary Fig. 1b). We used the median SUV value to determine the cut-off points for SUVmean-VAT and SUVmax-tumor as 0.41 and 3.30, respectively. According to the cutoff value, 73 (49.3%) and 75 (50.7%) patients were assigned to the high- and low SUVmean-VAT groups, respectively. No clinicopathological differences were observed between the high- and low SUVmean-VAT groups (Table 1).

**Table 1:** Clinical characteristics in relation to the ^18^F-FDG uptake of visceral adipose tissue.

	SUV-High, n = 73 (%)	SUV-Low, n = 75 (%)	*p* value
Age (year, mean ± SD)	50.90 ± 11.22	49.08 ± 8.30	0.264
BMI (mean ± SD)	23.19 ± 3.49	23.50 ± 3.11	0.571
SUVmax-tumor	4.50 ± 3.28	4.47 ± 4.04	0.960
Serum glucose (mg/dL, mean ± SD)	99.80 ± 14.18	103.24 ± 20.80	0.257
DM			0.513
No	71 (97.3)	72 (96.0)	
Yes	2 (2.7)	3 (4.0)	
ER			0.211
Positive	47 (64.4)	41 (54.7)	
Negative	23 (31.5)	31 (41.3)	
Missing	3 (4.1)	3 (4)	
PR			0.721
Positive	40 (54.8)	39 (52)	
Negative	30 (41.1)	33 (44)	
Missing	3 (4.1)	3 (4)	
HER2			0.657
Positive	17 (23.3)	21 (28)	
Negative	48 (65.8)	50 (66.7)	
Missing	8 (11.0)	4 (5.3)	
HG			0.942
I, II	39 (53.4)	38 (50.7)	
III	26 (35.6)	26 (34.7)	
Missing	8 (11.0)	11 (14.7)	
Tumor size			0.168
≤ 2 cm	48 (65.8)	41 (54.7)	
> 2 cm	25 (34.2)	34 (45.3)	
Lymph node metastasis			0.414
Negative	47 (64.4)	53 (70.7)	
Positive	26 (35.6)	22 (29.3)	
AJCC stage*			0.943
I	35 (47.9)	36 (48)	
II	30 (41.1)	32 (42.7)	
III	8 (11.0)	7 (9.3)	
Lymphovascular invasion			0.628
Negative	12 (16.4)	14 (18.7)	
Positive	52 (71.2)	49 (65.3)	
Missing	9 (12.3)	12 (16)	
Chemotherapy			0.765
Done	45 (61.6)	48 (64)	
Not done	25 (34.2)	24 (32)	
Missing	3 (4.1)	3 (4)	
Radiotherapy			0.203
Done	33 (45.2)	24 (32)	
Not done	37 (50.7)	42 (56)	
Missing	3 (4.1)	9 (12)	
Endocrine therapy			0.446
Done	49 (67.1)	44 (58.7)	
Not done	23 (31.5)	27 (36)	
Missing	1 (1.4)	4 (5.3)	

*SD* Standard deviation; *BMI* Body mass index; *DM* Diabetes mellitus; *ER* Estrogen receptor; *PR* Progesterone receptor; *HER*2, Human epidermal growth factor receptor 2; *HG* Histologic grade.

*AJCC stage was performed based on 8th edition.

### Prognostic significance of ^18^F-FDG uptake of VAT

**Figure 2:**
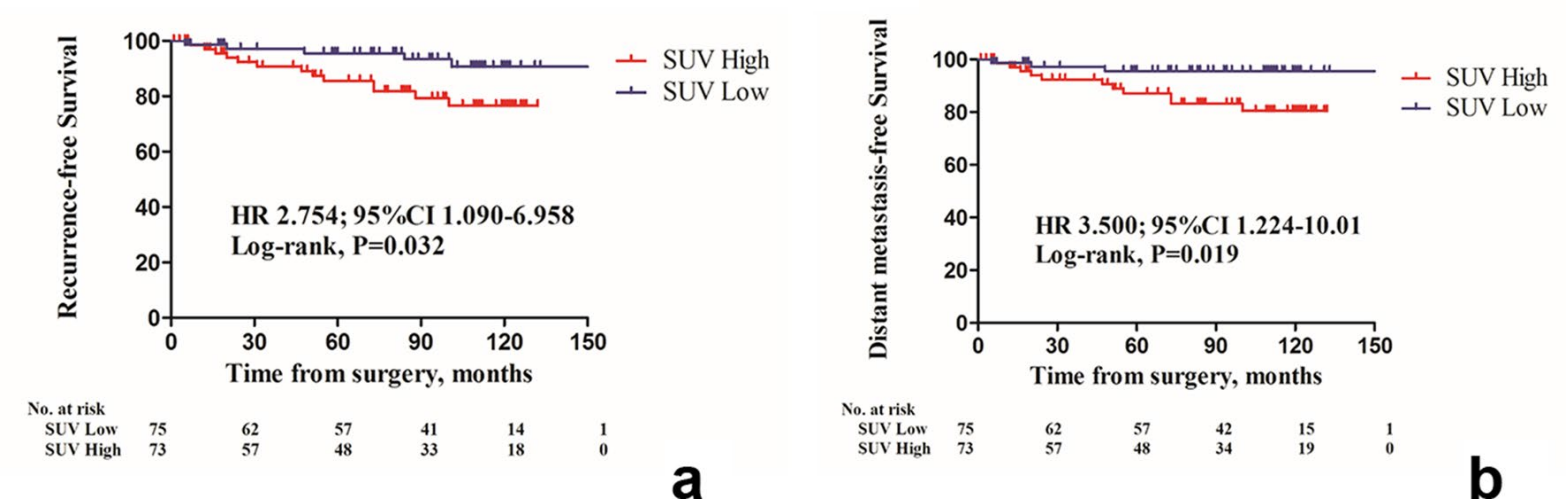
Kaplan–Meier survival curves of (**a**) recurrence-free survival (RFS) and (**b**) distant metastasis-free survival (DMFS) according to the ^18^F-FDG uptake of visceral adipose tissue (VAT). Patients with high SUVmean-VAT showed a poorer RFS and DMFS, (A, hazard ratio 2.754, 95% confidence interval [CI] 1.090–6.958, *p* = 0.032; B, HR 3.500, 95% CI 1.224–10.01, *p* = 0.019, respectively).

By the median follow-up duration of 95.5 months, recurrence and distant metastases had occurred in 18 and 14 patients, respectively. The median follow-up durations of the low SUVmax-tumor group and high SUVmax-tumor group were 96 months (range, 6–152 months) and 94 months (range, 1–132 months) for each. In the low SUVmax-tumor group, three patients had metastases in the liver, mediastinal lymph node, and contralateral axillary lymph node, respectively. In the high SUVmax-tumor group, 11 metastatic events that had occurred (including duplicates) at the following sites: bone (n = 4), lung (n = 2), cervical lymph node (n = 2), mediastinal lymph node (n = 2), liver (n = 1), pleura (n = 1), and intraperitoneal lymph node (n = 1). The high SUVmean-VAT group had significantly shorter recurrence-free survival (RFS) and distant metastasis-free survival (DMFS) (Fig. 2a; hazard ratio [HR], 2.754; 95% confidence interval [CI], 1.090–6.958; *p* = 0.032; Fig. 2b; HR, 3.500; 95% CI, 1.224–10.01; *p* = 0.019, respectively—compared using the log-rank test].

Regarding RFS, significant risk factors for RFS were lymph node metastasis, advanced American Joint Committee on Cancer (AJCC) stage, no endocrine therapy, and high SUVmean-VAT (Table 2; HR, 2.928; 95% CI, 1.043–8.215; *p* = 0.041). Among these, high SUVmean-VAT was a significant independent risk factor for shorter RFS in the multivariate analysis (Table 2; HR, 3.361; 95% CI, 1.181–9.566; *p* = 0.023).

**Table 2:** Hazard ratios (HRs) and 95% confidence intervals (CIs) for recurrence-free survival (RFS).

	Univariate analysis		Multivariate analysis	
	HRs (95% CIs)	*p* value	HRs (95% CIs)	*p* value
Age (continuous)	1.007 (0.957–1.059)	0.785	N/A	
BMI (continuous)	1.047 (0.920–1.191)	0.490	N/A	
ER		0.239	N/A	
Negative	1			
Positive	0.574 (0.228–1.446)			
PR		0.102	N/A	
Negative	1			
Positive	0.454 (0.176–1.171)			
HER2		0.704	N/A	
Negative	1			
Positive	0.806 (0.265–2.450)			
HG		0.864	N/A	
I, II	1			
III	1.044 (0.637–1.711)			
Tumor size		0.228	N/A	
≤ 2 cm	1			
> 2 cm	1.766 (0.701–4.450)			
Lymph node metastasis		0.037		0.597
Negative	1		1	
Positive	2.689 (1.061–6.815)		1.404 (0.398–4.954)	
AJCC stage		0.040		0.079
I	1		1	
II	3.745 (1.192–11.764)		3.197 (1.015–10.072)	
III	3.534 (0.791–15.798)		3.337 (0.745–14.935)	
Lymphovascular invasion		0.820	N/A	
Negative	1			
Positive	0.864 (0.246–3.039)			
SUVmean-VAT		0.041		0.023
Low	1		1	
High	2.928 (1.043–8.215)		3.361 (1.181–9.566)	
Chemotherapy		0.543	N/A	
Not done	1			
Done	0.745 (0.289–1.922)			
Radiotherapy		0.969	N/A	
Not done	1			
Done	0.982 (0.387–2.491)			
Endocrine therapy		0.021		0.014
Not done	1		1	
Done	0.329 (0.127–0.848)		0.300 (0.115–0.786)	

*BMI* Body mass index; *ER* Estrogen receptor; *PR* Progesterone receptor; *HER*2 Human epidermal growth factor receptor 2; *HG* Histologic grade; *VAT* Visceral adipose tissue; *N/A* Not assessed.

High SUVmean-VAT and advanced AJCC were significant risk factors for DMFS in the univariate and multivariate analyses (Table 3).

**Table 3:** Hazard ratios (HRs) and 95% confidence intervals (CIs) for distant metastasis-free survival (DMFS).

	Univariate analysis		Multivariate analysis	
	HRs (95% CIs)	*p* value	HRs (95% CIs)	*p* value
Age (continuous)	1.024 (0.968–1.082)	0.408	N/A	
BMI	1.005 (0.861–1.173)	0.949	N/A	
ER		0.119	N/A	
Negative	1			
Positive	0.569 (0.200–1.624)			
PR		0.098	N/A	
Negative	1			
Positive	0.397 (0.133–1.184)			
HER2		0.836	N/A	
Negative	1			
Positive	1.131 (0.355–3.605)			
HG		0.994	N/A	
I, II	1			
III	0.998 (0.562–1.772)			
Tumor size		0.119	N/A	
≤ 2 cm	1			
> 2 cm	2.320 (0.804–6.688)			
Lymph node metastasis		0.052	N/A	
Negative	1			
Positive	2.859 (0.992–8.241)			
AJCC stage		0.015		0.019
I	1		1	
II	6.024 (1.301–27.886)		5.810 (1.255–26.900)	
III	7.125 (1.190–42.650)		6.631 (1.106–39.734)	
Lymphovascular invasion		0.693	N/A	
Negative	1			
Positive	1.301 (0.352–4.815)			
SUVmean-VAT		0.031		0.039
Low	1		1	
High	4.066 (1.134–14.580)		3.846 (1.072–13.795)	
Chemotherapy		0.392	N/A	
Not done	1			
Done	0.630 (0.218–1.815)			
Radiotherapy		0.915	N/A	
Not done	1			
Done	0.944 (0.327–2.723)			
Endocrine therapy		0.082	N/A	
Not done	1			
Done	0.390 (0.135–1.125)			

*BMI* Body mass index; *ER* Estrogen receptor; *PR* Progesterone receptor; *HER*2 Human epidermal growth factor receptor 2; *HG* Histologic grade; *VAT* Visceral adipose tissue; *N/A* Not assessed.

### Prognostic significance of the ^18^F-FDG uptake of tumor

The patients were divided into high (n = 73, 49.3%) and low (n = 75, 50.7%) SUVmax-tumor groups. The high SUVmax-tumor group had the following aggressive features: estrogen receptor (ER)-negative, progesterone (PR)-negative, higher histologic grade (HG), larger tumor size, advanced AJCC stage, and no endocrine treatment (Supplementary Table 1).

Kaplan–Meier survival plots for RFS and DFMS were drawn to compare the high- and low SUVmax-tumor groups (Supplementary Fig. 2). The high SUVmax-tumor group had a significantly inferior RFS (Supplementary Fig. 2a; HR, 2.760; 95% CI, 1.092–6.974; *p* = 0.0388); however, there was no difference in DMFS between the two groups in relation to the SUVmax-tumor (Supplementary Fig. 2b; HR, 2.625; 95% CI, 0.918–7.509; *p* = 0.0718).

High SUVmax-tumor was a significant risk factor for shorter RFS in the univariate analysis (Supplementary Table 2; HR, 2.934; 95% CI, 1.046–8.232, *p* = 0.041). The high SUVmax-tumor group tended to have a shorter DMFS; however, the difference was not significant (Supplementary Table 2; HR, 2.772; 95% CI, 0.869–8.841; *p* = 0.085).

## Discussion

In this study, we measured both SUVmean-VAT and SUVmax-tumor using PET/CT in patients with breast cancer and found that high SUVmean-VAT was significantly associated with tumor recurrence. Our results suggest that activated visceral fat metabolism may be a potential clinical target for breast cancer treatment by controlling dysmetabolism and systemic inflammation.

Understanding where and how cellular energy consumption occurs in humans in vivo is the key to understanding cancer metabolism. Obesity and related metabolic complications are considered to have a clinical impact; this has triggered intense research to elucidate the comprehensive molecular-level mechanisms^10,11^. Glucose utilization in VAT is different from that in SAT in terms of inflammatory mediators, gene expression, and cell morphology^12–15^. Christen et al. found that VAT has a higher glucose uptake and different metabolic activity than non-visceral adipose tissue using ^18^F-FDG PET imaging^16^. Visceral adiposity is known to increase cancer risk^17,18^, and is thought to be a surrogate parameter for cancer incidence^19,20^. Strategies for fat remodeling (adipose tissue browning) and lifestyle interventions have been suggested^20,21^. These results illustrate that VAT might be involved in cancer metabolism and act as a biomarker, which is in line with our results of VAT being a significant prognostic marker in breast cancer.

In this study, there was no significant difference in clinicopathological characteristics between the high- and low SUVmean-VAT groups. Both RFS and DMFS were significantly inferior in the high SUVmean-VAT group (*p* = 0.032 and 0.019, respectively). Regarding overall survival, only five patients died during the study period, which was too small for an appropriate statistical analysis. However, there was a trend of increased death events in the high SUVmean-VAT group than in the low SUVmean-VAT group (n = 4 vs. n = 1), although the difference was not significant (*p* = 0.149). In the Cox proportional hazard model, SUVmean-VAT was found to be an independent predictor of shorter RFS and DMFS, even more significant than pathologic parameters, including tumor size and lymphovascular invasion (LVI).

To the best of our knowledge, this is the first study to report the prognostic impact of VAT metabolism measured using ^18^F-FDG PET/CT in patients with breast cancer. There was a clear association between high SUVmean-VAT and survival outcomes in patients with breast cancer. PET/CT is a preoperative non-invasive assessment modality that can evaluate the whole body during a single examination and provides additional information such as tumor metabolism, systemic inflammatory status, cancer diagnosis, and staging.

Many studies have investigated the effects of adipocyte biology on cytokine production^22–25^. Adipokines, secreted by adipocytes, have been receiving attention for their role in cancer metabolism^26^. Regarding VAT, a special compartment of adipocytes, its upregulated activity has been a point of focus in several studies: VAT activity was related to myocardial infarction^8^. It also reflected the inflammatory status of the carotid artery in metabolic syndrome^27,28^. The predictive role of VAT activity has been assessed by preoperative ^18^F-FDG PET/CT in gastric, colorectal, and thyroid cancers^29–31^. These studies reported that dysfunctional adipocytes in VAT secrete inflammatory adipokines that induce an inflammation-sustaining microenvironment, which in turn promotes carcinogenesis and tumor cell growth. Hence, the metabolic activity of VAT may be a surrogate marker for tumor progression, and glucose metabolism could be measured using ^18^F-FDG PET/CT.

Obesity and obesity-driven metabolic dysfunction in postmenopausal women are risk factors for breast cancer^32^. A recent study of 173 postmenopausal patients with breast cancer showed that the SUV ratio of the VAT and SAT correlated significantly with that of axillary lymph node metastasis^9^. This is partially in line with our finding of the association between high SUVmean-VAT and the biologically aggressive behavior of breast cancer. White adipose tissue, which comprises most VAT, has an increased number of mast cells^33^ and macrophages^34^ that secrete pro-inflammatory cytokines. The systemic release of pro-inflammatory cytokines induces chronic low-grade inflammation, which results in the production of reactive oxygen species and enhancement of tumor promotion^35^. A previous study found that VAT, but not SAT, was independently associated with upregulated serum inflammatory markers including white blood cell count and high-sensitivity CRP^36^. Furthermore, VAT is more metabolically active and has a larger number of inflammatory and immune cells, which confers its greater glucose uptake capacity than SAT^37^. Thus, SUVmean-VAT may be a non-invasive marker of systemic inflammation and a prognostic indicator in patients with breast cancer.

This study has several limitations. First, this study had a retrospective design, a small sample size, and was a single-center study. Although significant prognoses were observed, in-depth analyses of the patients’ characteristics (e.g., post-menopause) or characteristics of the molecular subgroups were not performed. Second, we included all available breast cancers for analysis regardless of the molecular subtype, which might have resulted in an uneven distribution of patients in each molecular subgroup and selection bias that could skew the statistics. Third, SUVmean-VAT, the main parameter analyzed in this study, had a very narrow range of values; this implies that SUVmean-VAT might be vulnerable to changes in other variables during PET/CT procedures, such as scanning, imaging protocols, radiopharmaceutical dose, and image acquisition time after ^18^F-FDG injection. These variables should be controlled precisely for an accurate SUV acquisition^37,38^. Nonetheless, SUV is affected by multiple factors, such as plasma glucose and insulin levels; thus, inter-subject and intra-subject variability could also exist. Further validation of these findings in prospective large multicenter studies and other types of malignancies is required. However, despite these limitations, we observed a significant correlation between SUVmean-VAT and patient prognosis in breast cancer.

In conclusion, the increased glucose metabolism of VAT assessed using ^18^F-FDG PET/CT was significantly associated with RFS and DMFS in patients with breast cancer, which might also reflect pro-tumor systemic inflammation. Upregulated SUVmean-VAT levels in patients with breast cancer could be a predictor of tumor recurrence.

## Methods

### Patients

This was a retrospective study. Patients with invasive breast cancer (stages I–III; age ≥ 20 years at the time of surgery) who were treated between January 2007 and December 2010 at Gangnam Severance Hospital according to standard protocols were included in the study. The patients were followed up till December 31, 2020. We collected the patients’ clinical information from their electronic medical records, including data on age, body mass index (BMI), treatment modalities (chemotherapy, radiotherapy, and endocrine therapy), and survival outcomes including recurrence and metastasis. RFS was defined as the interval from the date of primary surgery to the date of any breast cancer recurrence (locoregional and/or distant metastasis), death due to any cause, or last follow-up. DMFS was defined as the interval from primary curative surgery to the first breast cancer-derived distant metastasis, death due to any cause, or end of follow-up. Data of patients who did not exhibit relevant events were censored at the end of the follow-up period. The patients’ pathological parameters were collected from pathologic reports of resected specimens. These included tumor size, HG, LVI, lymph node metastasis, and status of ER, PR, and human epidermal growth factor receptor 2. The anatomical tumor (T), node (N), and metastasis (M) (TNM) stages were classified according to the AJCC 8th edition^39^. The histologic grade of the tumor was determined according to the modified Scarf–Bloomer–Richardson grading system^40^. Patients who met the following criteria were excluded:

1. Only ductal carcinoma in situ
2. Stage IV breast cancer
3. Other malignancies (except for thyroid cancer)
4. Unavailable electronic medical records
5. Unavailable detailed PET/CT information
6. Other inflammatory diseases (including autoimmune diseases and infections)
7. Organ failure
8. PET/CT image from the referral hospital with different protocols

The patients were categorized into high and low groups according to their median SUVmean-VAT and SUVmax-tumor (high SUVmean-VAT /low SUVmean-VAT and high SUVmax-tumor/low SUVmax-tumor groups).

This study was approved by the Institutional Review Board (IRB) of Gangnam Severance Hospital (Local IRB Number: 3-2021-0074) in Seoul, Republic of Korea. The IRB waived the requirement for informed consent due to the retrospective study design.

### ^18^F-FDG PET/CT acquisition

All the patients fasted for at least 6 h and had blood glucose levels below 140 mg/dL before ^18^F-FDG (dose: 5.5 MBq/kg of body weight) was intravenously administered, and ^18^F-FDG PET/CT was performed using a PET/CT scanner (Biograph 40 TruePoint, Siemens Healthcare Solutions USA, Inc., Knoxville, TN). Whole-body low-dose CT images were obtained for attenuation correction using automatic dose modulation against the standard reference values of 40 mA and 120 kV. The CT images were acquired with a 3 mm slice thickness and up to 4640 projections per 360° rotations. The scanned images were reconstructed onto 512 × 512-pixel CT image matrices (0.98 mm × 0.98 mm × 3.0 mm) using the B30f. kernel. PET data were acquired from the skull base to the proximal thigh for 3 min per bed position in the three-dimensional mode. PET images were reconstructed using the ordered subset expectation maximization algorithm (OSEM) using two iterations and 21 subsets. The matrix size and thickness of the reconstructed PET images were 128 × 128 and 5 mm, respectively. Automated corrections such as normalization, correction for random coincidences, correction for scattered radiation, and correction for dead time were applied. Maximum intensity projection (MIP), cross-sectional views, and fusion images were generated and reviewed. Cross-calibration between PET and the dose calibrator was performed monthly.

### PET/CT image analysis and data acquisition of SUV

All PET/CT images were evaluated by experienced nuclear medicine physicians, who were blinded to the participant’s clinical data. Decisions concerning analyses of the PET/CT datasets were reached by consensus. Image analysis was performed using the MIM software (MIM, 6.8.10, Cleveland, OH, USA). For semi-quantitative evaluation, the SUV was calculated by measuring the tumor absorption of ^18^F-FDG in the region of interest (ROI) as follows:

$${\text{SUV}} = \left[ {\text{Radioactivity concentration in the ROI}} \right]/\left[ {{\text{Injected dose}}/{\text{patient weight }}\left( {{\text{kg}}} \right)} \right]$$

**Figure 3:**
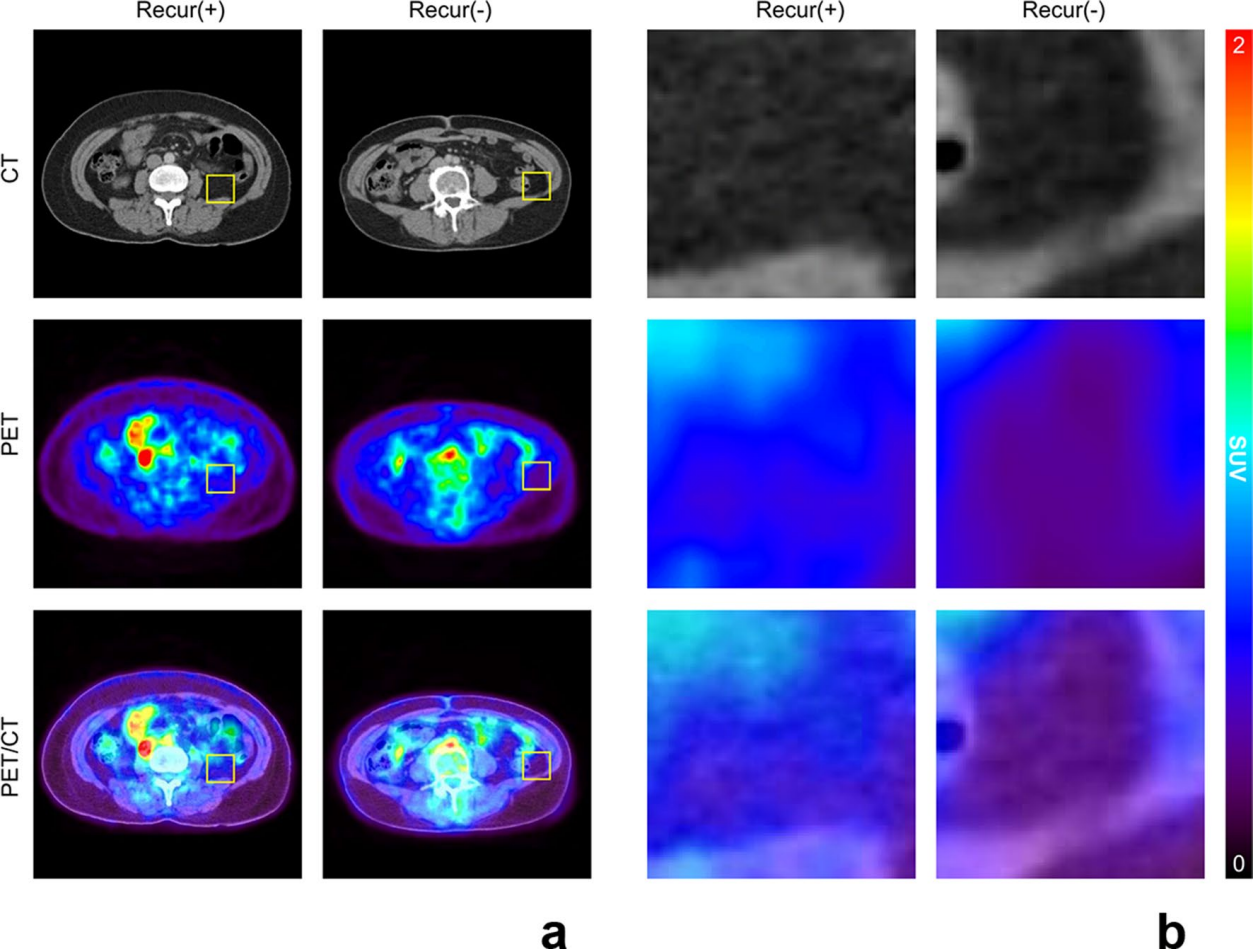
Representative images of (**a**) ^18^F-FDG uptake of visceral adipose tissue (VAT) according to the breast cancer recurrence status and (**b**) their corresponding magnified views. CT: computed tomography, PET: positron emission tomography, Recur: Recurrence.

A volume of interest (VOI) was manually drawn over the primary breast cancer lesion on the PET/CT images, and the SUVmax-tumor was measured. The image analysis was performed to assess the metabolic activity of VAT. The VAT regions were recognized based on predefined Hounsfield units (range: − 110 to − 70 HU^41^, which corresponds to adipose tissue densities in humans) from the corresponding CT images, as described previously^9,16,27,42^. There are several methods to measure the SUV for VAT. The method of measuring the SUV in multiple consecutive planes and calculating the average was widely used in previous studies and can reduce the bias or noise that occurs when only one ROI is selected. Regarding the evaluation of VAT uptake, two-dimensional ROIs were placed on three consecutive slices of the abdominal VAT area. Furthermore, ROIs were placed away from the intestines, kidneys, vessels, and/or muscles, to exclude the inclusion of physiological ^18^F-FDG uptake in these areas^9,27,30,42^. We obtained the mean SUV from consecutive ROIs. Both intra—and interobserver correlation analyses in SUV measurements demonstrated excellent reliability (correlation coefficient > 0.9). The differences in the glucose uptake of VAT according to breast cancer recurrence on PET-CT are shown in Fig. 3.

The interval time between PET/CT acquisition and curative surgery was 3.79 ± 5.01 days in 145 patients, excluding 4 patients who received neoadjuvant chemotherapy.

### Statistical analysis

Continuous variables between the two groups were compared using Student’s t-test or Mann–Whitney U test. Categorical variables were compared using the chi-square or Fisher’s exact test. Survival curves were obtained using the Kaplan–Meier method, and two-group comparisons were performed using the log-rank test. Univariate and multivariate Cox proportional hazard models were used to identify the factors associated with survival outcomes (RFS and DMFS). Variables that were significant in the univariate analysis were included in the multivariate Cox proportional hazards models. Statistical analyses were performed using SPSS version 24 (SPSS Inc., Chicago, IL, USA). The threshold for statistical significance was set at *p* < 0.05, with a 95% confidence interval (CI) not including 1.

### Ethics approval and consent to participate

All procedures performed in studies involving human participants were in accordance with the ethical standards of the institutional and/or national research committee and with the 1964 Declaration of Helsinki and its later amendments or comparable ethical standards. The protocol was approved by the Institutional Review Board (Local IRB Number: 3-2021-0074) of Gangnam Severance Hospital. The need for informed consent was waived by the IRB due to the retrospective study design.

## Supplementary Information

Supplementary Information 1.

Supplementary Information 2.

Supplementary Information 3.

## Data Availability

All data generated or analyzed during this study are included in this research article and the supplementary information files.

## Abbreviations

CRP: C-reactive protein
SIP: Sphingosine 1-phosphate
^18^F-FDG: ^18^Fluorodeoxyglucose
PET: Positron emission tomography
CT: Computed tomography
VAT: Visceral adipose tissue
SAT: Subcutaneous adipose tissue
SUVmax: Maximum standardized uptake value
BMI: Body mass index
HG: Histologic grade
ER: Estrogen receptor
PR: Progesterone receptor
HER2: Human epidermal growth factor receptor 2
LVI: Lymphovascular invasion
TNM: Tumor, node, metastasis
AJCC: American joint committee on cancer
IRB: Institutional review board
OSEM: Ordered subset expectation maximization algorithm
MIP: Maximum intensity projection
ROI: Region of interest
VOI: Volume of interest
RFS: Recurrence-free survival
DMFS: Distant metastasis-free survival
HR: Hazard ratio
CI: Confidence interval

## References

[1] Jemal A (2007). Cancer statistics, 2007. CA Cancer J. Clin..

[2] McAndrew NP (2021). Effects of systemic inflammation on relapse in early breast cancer. NPJ Breast Cancer.

[3] Ham M, Moon A (2013). Inflammatory and microenvironmental factors involved in breast cancer progression. Arch. Pharmacal. Res..

[4] Edsparr K, Basse PH, Goldfarb RH, Albertsson P (2011). Matrix metalloproteinases in cytotoxic lymphocytes impact on tumour infiltration and immunomodulation. Cancer Microenviron. Off. J. Int. Cancer Microenviron. Soc..

[5] Kessenbrock K, Plaks V, Werb Z (2010). Matrix metalloproteinases: Regulators of the tumor microenvironment. Cell.

[6] Long JS (2010). Sphingosine kinase 1 induces tolerance to human epidermal growth factor receptor 2 and prevents formation of a migratory phenotype in response to sphingosine 1-phosphate in estrogen receptor-positive breast cancer cells. Mol. Cell. Biol..

[7] Kim ES (2014). Inflammatory lipid sphingosine-1-phosphate upregulates C-reactive protein via C/EBPbeta and potentiates breast cancer progression. Oncogene.

[8] Pahk K, Kim EJ, Joung C, Seo HS, Kim S (2020). Association of glucose uptake of visceral fat and acute myocardial infarction: A pilot (18)F-FDG PET/CT study. Cardiovasc. Diabetol..

[9] Pahk K, Joung C, Kim S (2020). Visceral fat metabolic activity evaluated by preoperative (18)F-FDG PET/CT significantly affects axillary lymph node metastasis in postmenopausal luminal breast cancer. Sci. Rep..

[10] Atawia RT, Bunch KL, Toque HA, Caldwell RB, Caldwell RW (2019). Mechanisms of obesity-induced metabolic and vascular dysfunctions. Front. Biosci. (Landmark Ed).

[11] Jung UJ, Choi MS (2014). Obesity and its metabolic complications: The role of adipokines and the relationship between obesity, inflammation, insulin resistance, dyslipidemia and nonalcoholic fatty liver disease. Int. J. Mol. Sci..

[12] Perrini S (2008). Fat depot-related differences in gene expression, adiponectin secretion, and insulin action and signalling in human adipocytes differentiated in vitro from precursor stromal cells. Diabetologia.

[13] Virtanen KA (2005). Increased fat mass compensates for insulin resistance in abdominal obesity and type 2 diabetes: A positron-emitting tomography study. Diabetes.

[14] Virtanen KA (2002). Glucose uptake and perfusion in subcutaneous and visceral adipose tissue during insulin stimulation in nonobese and obese humans. J. Clin. Endocrinol. Metab..

[15] Westergren H, Danielsson A, Nystrom FH, Stralfors P (2005). Glucose transport is equally sensitive to insulin stimulation, but basal and insulin-stimulated transport is higher, in human omental compared with subcutaneous adipocytes. Metab. Clin. Exp..

[16] Christen T (2010). Increased glucose uptake in visceral versus subcutaneous adipose tissue revealed by PET imaging. JACC Cardiovasc. Imaging.

[17] Renehan AG, Tyson M, Egger M, Heller RF, Zwahlen M (2008). Body-mass index and incidence of cancer: A systematic review and meta-analysis of prospective observational studies. Lancet.

[18] Calle EE, Rodriguez C, Walker-Thurmond K, Thun MJ (2003). Overweight, obesity, and mortality from cancer in a prospectively studied cohort of U.S. adults. N. Engl. J. Med..

[19] Okamura T (2020). Visceral adiposity index is a predictor of incident colorectal cancer: A population-based longitudinal study. BMJ Open Gastroenterol..

[20] Crudele L, Piccinin E, Moschetta A (2021). Visceral adiposity and cancer: Role in pathogenesis and prognosis. Nutrients.

[21] Kurylowicz A, Puzianowska-Kuznicka M (2020). Induction of adipose tissue browning as a strategy to combat obesity. Int. J. Mol. Sci..

[22] Despres JP, Lemieux I (2006). Abdominal obesity and metabolic syndrome. Nature.

[23] Fuster JJ, Ouchi N, Gokce N, Walsh K (2016). Obesity-induced changes in adipose tissue microenvironment and their impact on cardiovascular disease. Circ. Res..

[24] Horakova D (2018). Total and high-molecular-weight adiponectin levels and prediction of insulin resistance. Endokrynol. Pol..

[25] Tchernof A, Despres JP (2013). Pathophysiology of human visceral obesity: An update. Physiol. Rev..

[26] Choi J, Cha YJ, Koo JS (2018). Adipocyte biology in breast cancer: From silent bystander to active facilitator. Prog. Lipid Res..

[27] Bucerius J (2015). Impact of bariatric surgery on carotid artery inflammation and the metabolic activity in different adipose tissues. Medicine (Baltimore).

[28] Tahara N (2007). Vascular inflammation evaluated by 18F-fluorodeoxyglucose positron emission tomography is associated with the metabolic syndrome. J. Am. Coll. Cardiol..

[29] Lee JW (2020). Significance of CT attenuation and F-18 fluorodeoxyglucose uptake of visceral adipose tissue for predicting survival in gastric cancer patients after curative surgical resection. Gastric Cancer.

[30] Pahk K, Choi S, Kim S (2018). Functional visceral fat activity evaluated by preoperative F-18 FDG PET/CT predicts regional lymph node metastasis in differentiated thyroid cancer. Clinical Endocrinology.

[31] Pahk K, Rhee S, Kim S, Choe JG (2016). Predictive role of functional visceral fat activity assessed by preoperative F-18 FDG PET/CT for regional lymph node or distant metastasis in patients with colorectal cancer. PLoS ONE.

[32] Park B, Kim S, Kim H, Cha C, Chung MS (2021). Associations between obesity, metabolic health, and the risk of breast cancer in East Asian women. Br. J. Cancer.

[33] Mukai K, Tsai M, Saito H, Galli SJ (2018). Mast cells as sources of cytokines, chemokines, and growth factors. Immunol. Rev..

[34] Cancello R (2006). Increased infiltration of macrophages in omental adipose tissue is associated with marked hepatic lesions in morbid human obesity. Diabetes.

[35] Sullivan, N. J. Interleukin-6 in the breast tumor microenvironment. In *Breast Cancer—Focusing Tumor Microenvironment, Stem Cells and Metastasis* (ed. M Gunduz), 165–175 (2011).

[36] Yu JY, Choi WJ, Lee HS, Lee JW (2019). Relationship between inflammatory markers and visceral obesity in obese and overweight Korean adults: An observational study. Medicine (Baltimore).

[37] Ibrahim MM (2010). Subcutaneous and visceral adipose tissue: Structural and functional differences. Obes. Rev..

[38] Thie JA (2004). Understanding the standardized uptake value, its methods, and implications for usage. J. Nucl. Med..

[39] Amin, M. B. E. S. B. G. F. L. A. J. C. o. C. AJCC cancer staging manual (2017).

[40] Elston CW, Ellis IO (1991). Pathological prognostic factors in breast cancer. I. The value of histological grade in breast cancer: Experience from a large study with long-term follow-up. Histopathology.

[41] Yoshizumi T (1999). Abdominal fat: Standardized technique for measurement at CT. Radiology.

[42] Vanfleteren LEGW (2014). A possible link between increased metabolic activity of fat tissue and aortic wall inflammation in subjects with COPD. A retrospective 18F-FDG-PET/CT pilot study. Respir. Med..

