# Supplementary figures and images for: 18F-FDG uptake of visceral adipose tissue on preoperative PET/CT as a predictive marker for breast cancer recurrence
Source: Sci Rep. 2022 Dec 6;12:21109. doi: 10.1038/s41598-022-25540-4 (PMC9727140; doi:10.1038/s41598-022-25540-4)

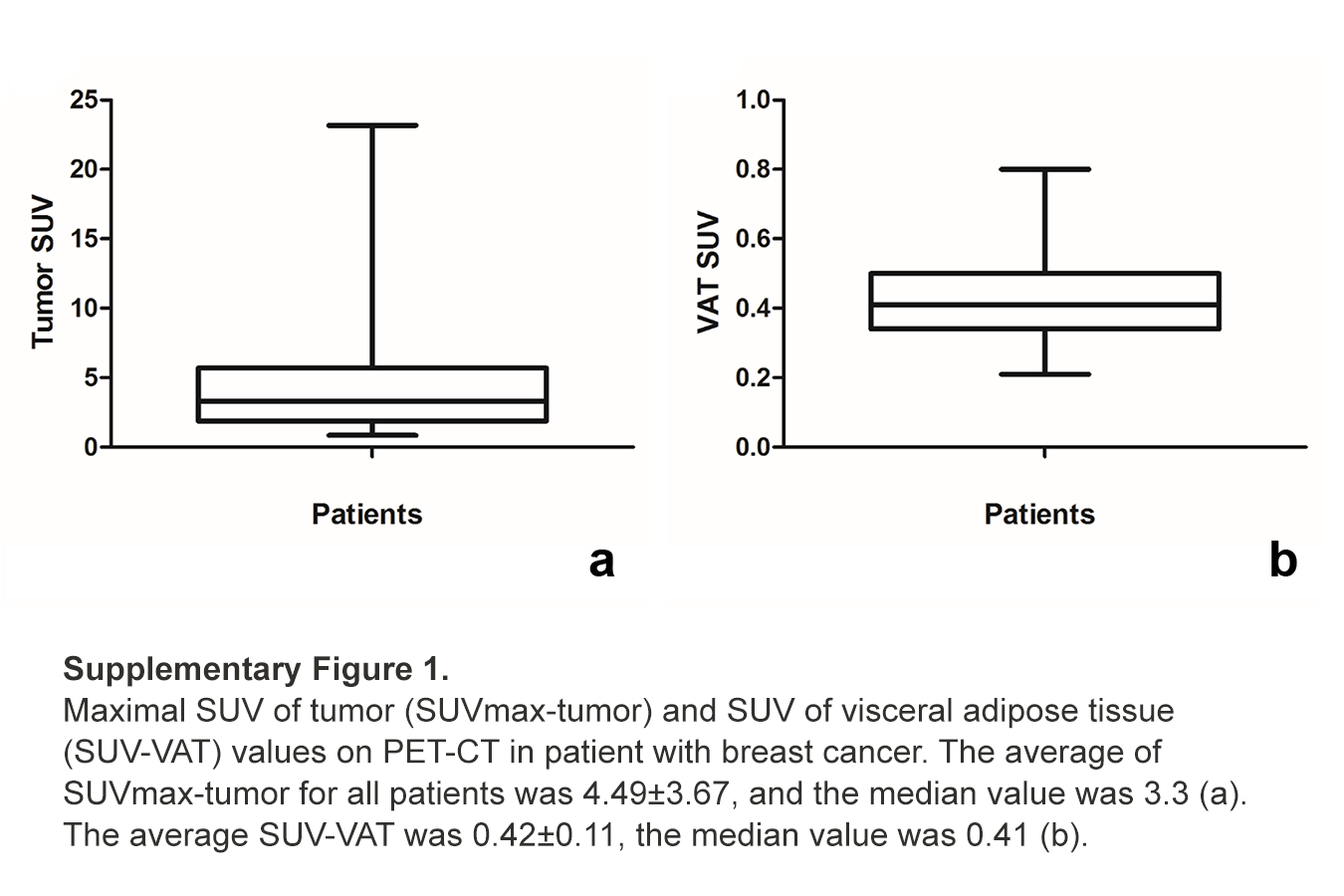

Supplement: Supplementary file 1 — Supplementary Information 1. [file 41598_2022_25540_MOESM1_ESM.tif]

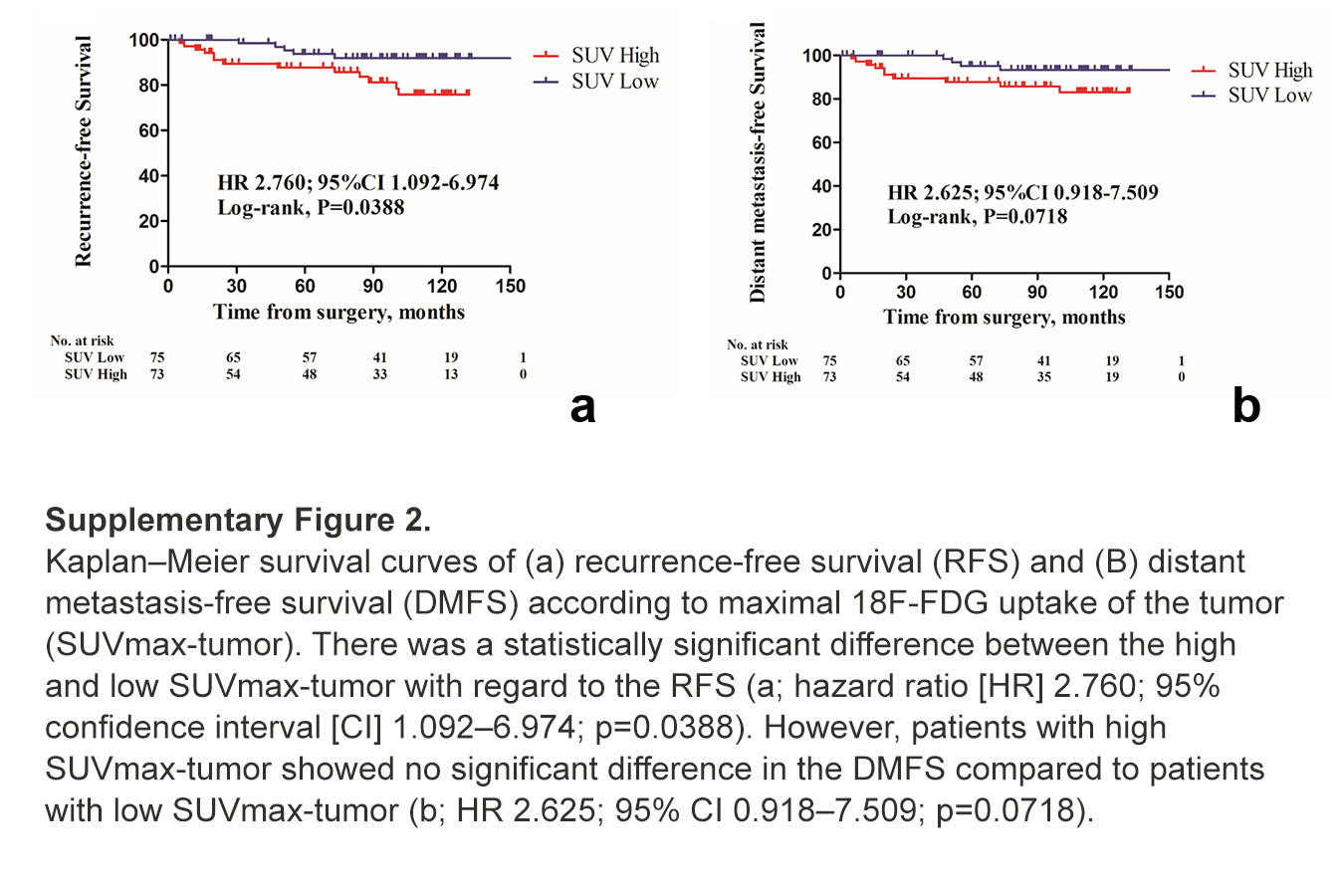

Supplement: Supplementary file 2 — Supplementary Information 2. [file 41598_2022_25540_MOESM2_ESM.tif]
