# Supplementary material for: 18F-FDG uptake of visceral adipose tissue on preoperative PET/CT as a predictive marker for breast cancer recurrence
Source: Sci Rep. 2022 Dec 6;12:21109. doi: 10.1038/s41598-022-25540-4 (PMC9727140; doi:10.1038/s41598-022-25540-4)
Supplement: Supplementary file 3 — Supplementary Information 3. [file 41598_2022_25540_MOESM3_ESM.docx]

**Supplementary Table 1.** Clinical characteristics in relation to the maximal ^18^F-FDG uptake of tumor

|  | **SUV-High, n=73 (%)** | **SUV-Low, n=75 (%)** | ***p* value** |
| --- | --- | --- | --- |
| **Age (year, mean±SD)** | 48.62±10.22 | 51.31±9.37 | 0.097 |
| **BMI (mean±SD)** | 23.64±3.80 | 23.06±2.71 | 0.286 |
| **SUV-VAT** | 0.43±0.12 | 0.41±0.11 | 0.435 |
| **Serum glucose (mg/dL, mean±SD)** | 101.19±15.63 | 101.99±19.90 | 0.788 |
| **DM** |  |  | 0.175 |
| **No** | 69 (94.5) | 74 (98.7) |  |
| **Yes** | 4 (5.5) | 1 (1.3) |  |
| **ER** |  |  | 0.003 |
| **Positive** | 36 (49.3) | 52 (69.3) |  |
| **Negative** | 36 (49.3) | 18 (24.0) |  |
| **Missing** | 1 (1.4) | 5 (6.7) |  |
| **PR** |  |  | 0.041 |
| **Positive** | 34 (46.6) | 45 (60) |  |
| **Negative** | 38 (52.1) | 25 (33.3) |  |
| **Missing** | 1 (1.4) | 5 (6.7) |  |
| **HER-2** |  |  | 0.188 |
| **Positive** | 15 (20.5) | 23 (30.7) |  |
| **Negative** | 51 (69.9) | 47 (62.7) |  |
| **Missing** | 7 (9.6) | 5 (6.7) |  |
| **HG** |  |  | <0.001 |
| **I, II** | 28 (38.4) | 49 (65.3) |  |
| **III** | 40 (54.8) | 12 (16) |  |
| **Missing** | 5 (6.8) | 14 (18.7) |  |
| **Tumor size** |  |  | 0.008 |
| **≤2 cm** | 36 (49.3) | 53 (70.7) |  |
| **>2 cm** | 37 (50.7) | 22 (29.3) |  |
| **Lymph node metastasis** |  |  | 0.129 |
| **Negative** | 45 (61.6) | 55 (73.3) |  |
| **Positive** | 28 (38.4) | 20 (26.7) |  |
| **AJCC stage^*^** |  |  | 0.015 |
| **I** | 27 (37.0) | 44 (58.7) |  |
| **II** | 35 (47.9) | 27 (36.0) |  |
| **III** | 11 (15.1) | 4 (5.3) |  |
| **Lymphovascular invasion** |  |  | 0.236 |
| **Negative** | 49 (67.1) | 52 (69.3) |  |
| **Positive** | 16 (21.9) | 10 (13.3) |  |
| **Missing** | 8 (11.0) | 13 (17.3) |  |
| **Chemotherapy** |  |  | 0.039 |
| **Done** | 53 (72.6) | 40 (53.3) |  |
| **Not done** | 19 (26.0) | 30 (40) |  |
| **Missing** | 1 (1.4) | 5 (6.7) |  |
| **Radiotherapy** |  |  | 0.385 |
| **Done** | 31 (42.5) | 26 (34.7) |  |
| **Not done** | 37 (50.7) | 42 (56) |  |
| **Missing** | 5 (6.8) | 7 (9.3) |  |
| **Endocrine therapy** |  |  | 0.004 |
| **Done** | 33 (45.2) | 55 (73.3) |  |
| **Not done** | 38 (52.1) | 17 (22.7) |  |
| **Missing** | 2 (2.7) | 3 (4) |  |

SD, standard deviation; BMI, body mass index; ER, estrogen receptor; PR, progesterone receptor; HER-2, human epidermal growth factor receptor-2; HG, histological grade

*AJCC stage was performed based on 8^th^ edition

**Supplementary Table 2. Evaluation of Cox proportional hazard model by ^18^F-FDG uptake of tumor**

|  | **RFS** | | **DMFS** | |
| --- | --- | --- | --- | --- |
|  | **HRs (95% CIs)** | ***p* value** | **HRs (95% CIs)** | ***p* value** |
| **SUVmax-tumor** |  | 0.041 |  | 0.085 |
| **Low** | 1 |  | 1 |  |
| **High** | 2.934 (1.046-8.232) |  | 2.772 (0.869-8.841) |  |

RFS, recurrence-free survival; DMFS, distant metastasis-free survival
